# A remote EPID-based dosimetric TPS-planned audit of centers for clinical trials: outcomes and analysis of contributing factors

**DOI:** 10.1186/s13014-018-1125-8

**Published:** 2018-09-17

**Authors:** Narges Miri, Kimberley Legge, Kim Colyvas, Joerg Lehmann, Philip Vial, Alisha Moore, Monica Harris, Peter B. Greer

**Affiliations:** 1 University of Newcastle, Newcastle, NSW Australia; 2 Calvary Mater Newcastle, Newcastle, NSW Australia; 3 Liverpool and Macarthur Cancer Therapy Centres and Ingham Institute, Liverpool, NSW Australia; 4 University of Sydney, Sydney, NSW Australia; 5 TROG Cancer Research, Newcastle, NSW Australia

**Keywords:** Auditing, Dosimetry, Electronic portal imaging device

## Abstract

**Background:**

A novel remote method for external dosimetric TPS-planned auditing of intensity modulated radiotherapy (IMRT) and volumetric modulated arc therapy (VMAT) for clinical trials using electronic portal imaging device (EPID) has been developed. The audit has been applied to multiple centers across Australia and New Zealand. This work aims to assess the audit outcomes and explores the variables that contributed to the audit results.

**Methods:**

Thirty audits were performed of 21 radiotherapy facilities, 17 facilities underwent IMRT audits and 13 underwent VMAT audits. The assessment was based on comparisons between the delivered doses derived from images acquired with EPIDs and planned doses from the local treatment planning systems (TPS). Gamma pass-rate (GPR) and gamma mean value (GMV) were calculated for each IMRT field and VMAT arc (total 268 comparisons). A multiple variable linear model was applied to the GMV results (3%/3 mm criteria) to assess the influence and significance of explanatory variables. The explanatory variables were Linac-TPS combination, TPS grid resolution, IMRT/VMAT delivery, age of EPID, treatment site, record and verification system (R&V) type and dose-rate. Finally, the audit results were compared with other recent audits by calculating the incidence ratio (IR) as a ratio of the observed mean/median GPRs for the remote audit to the other audits.

**Results:**

The average (± 1 SD) of the centers’ GPRs were: 99.3 ± 1.9%, 98.6 ± 2.7% & 96.2 ± 5.5% at 3%, 3 mm, 3%, 2 mm and 2%, 2 mm criteria respectively. The most determinative variables on the GMVs were Linac-TPS combination, TPS grid resolution and IMRT/VMAT delivery type. The IR values were 1 for seven comparisons, indicating similar GPRs of the remote audit with the reference audits and > 1 for four comparisons, indicating higher GPRs of the remote audit than the reference audits.

**Conclusion:**

The remote dosimetry audit method for clinical trials demonstrated high GPRs and provided results comparable to established more resource-intensive audit methods. Several factors were found to influence the results including some effect of Linac-TPS combination.

**Electronic supplementary material:**

The online version of this article (10.1186/s13014-018-1125-8) contains supplementary material, which is available to authorized users.

## Background

Starting in the mid-1990s, multileaf collimators (MLCs) were introduced to linear accelerators (linacs) to deliver a highly conformal dose to the patients. Inverse planning algorithms were added to treatment planning systems (TPSs) to plan the delivered dose when MLCs were used to modulate the profiles of beams. Intensity modulated beams formed the foundation of intensity modulated radiotherapy (IMRT) and volumetric modulated arc therapy (VMAT) deliveries [1]. Machine and patient specific quality assurance (QA) measurements are taken by local physicists to ensure accuracy and stability of IMRT/VMAT deliveries. The European Society for Radiotherapy and Oncology (ESTRO) recommends an additional external audit for independent verification [2]. Additionally, in the context of clinical trials, a dosimetry audit provides a controlled environment to minimize dependency of the outcome on stochastic and systematic errors that can reduce the trial cost and enhance the outcome reliability [3]. Conventionally, an auditing center performs the assessment by site visit(s) or by mailing phantoms and dosimeters [4, 5].

Remote auditing can significantly reduce the audit costs while enhancing the efficiency. Recently, a novel approach was introduced to remotely audit IMRT and VMAT deliveries [6, 7]. The method is termed the Virtual EPID Standard Phantom Audit (VESPA) and it is based on images from electronic portal imaging devices (EPIDs) and image to dose conversion models [8–11]. In VESPA, the audit center provides instructions and CT data for participants to produce benchmarking plans using their local TPS. These plans are then transferred to two provided virtual water phantoms and the doses exported. The participants deliver the dose in air to their EPID and send the corresponding images together with calibration images and their planning data to the audit center. The image signals are converted to dose in the virtual phantoms using in-house developed software. The method combines the cost and efficiency benefits of remote audits with a standardized measurement and analysis process using EPIDs. Details of the method and feasibility of the approach have been reported in a pilot study for six centers [7].

This work aims to assess the VESPA audit outcomes and explores the contribution of several explanatory variables to the overall outcomes of the audit. Results are presented for 30 audits from 21 treatment centers in terms of gamma analysis for multiple criteria. A multi-variable model was developed to understand whether the audit was sensitive to differences in equipment of the centers or other factors. Finally, the audit outcome was compared with other recent audits to assess whether the VESPA audit is consistent with conventional audit approaches.

## Methods

### Equipment

Participants were radiotherapy centers from Australia and New Zealand who were already treating patients with IMRT or VMAT and required credentialing for clinical trials by the Trans-Tasman Radiation Oncology Group (TROG). Additional file 1 provides details of the centers and their planning and treatment equipment. Of the 21 centers, 17 participated in the IMRT audit and 13 in the VMAT audit. TROG supplied a head and neck (HN) and a post-prostatectomy (PP) trial benchmarking plan case including CT datasets and planning instructions (TROG trials 12.01 HPV and 08.03 RAVES). Additionally CT datasets of two standard virtual water-equivalent QA phantoms were also provided; a virtual flat phantom (VFP) of 30 cm height, 40 cm width, 40 cm length and a virtual cylindrical phantom (VCP) of 20 cm diameter and 40 cm length. A separate EPID guide was included in the audit instructions to assist with calibration and data acquisition. As centers either submitted one or two plans for their audit, a total of 27 IMRT plans and 19 VMAT plans were submitted resulting in 268 individual IMRT fields or VMAT arcs.

### Planning and measurements

Each center planned the HN and PP trial patients on the provided patient datasets for IMRT or VMAT following the benchmarking instructions. A dose of 70 Gy was prescribed in 35 fractions for the HN plan and 64 Gy in 32 fractions for the PP plan. Except for one case at 10 MV these all were planned and delivered at 6 MV energy. The plans were then transferred onto the two supplied virtual phantoms within the local planning system. For 2D planar dose calculations the individual IMRT fields and VMAT arcs were transferred to the VFP at perpendicular incidence (zero gantry angle). This required collapsing all gantry angles to zero for the VMAT plans. For calculation of composite 3D dose the plans were transferred to the VCP at actual gantry angles. The phantoms were positioned at 90 cm source to surface distance (SSD). These verification plan doses were then exported in DICOM format. A DICOM-RT format TPS plan was also provided for calibration purposes.

All EPID measurements were made in-air with no phantom or treatment couch present. For the IMRT audit an integrated image for each field was acquired both at gantry zero and at actual gantry angles. For the VMAT audit EPID cine-images with 5 frames averaged per image were acquired continuously throughout the delivery of each arc and the acquired angle for each cine-image was recorded. These were summed to obtain an integrated image for each arc. A calibration plan was also provided to determine EPID positioning and sag with gantry angle as well as to calibrate EPID signal to dose. The centers exported their images and TPS doses and uploaded them via the cloud to the auditing site for assessment.

### Analysis

All analysis was performed by the auditing site using in-house software developed in MATLAB (The Mathworks, Natick, USA). Integrated images of each individual IMRT field and VMAT arc delivery were used to reconstruct 2D dose planes at 10 cm depth in the VFP. Details of the method to calculate dose in phantom from EPID images have been detailed previously [7, 8]. For calculation of composite 3D dose in the VCP, a similar method to Ansbacher [12] was used with the IMRT images at actual gantry angles and the cine images for VMAT delivery. These were converted to dose in the VCP using the same dose conversion model as for the 2D individual field analysis but with additional contour correction and percentage depth dose modelling to derive 3D dose.

An in-house developed gamma (*γ*) algorithm was used for the dose comparison. All doses above 10% of the maximum dose were assessed with a search region of 0.6 cm radius. The gamma function used a global dose difference criteria defined as a percentage of the maximum dose. For 2D dose planes from individual fields or arcs, 2D gamma analysis was employed with the TPS dose map interpolated to the EPID resolution. Gamma pass-rate (GPR) and gamma mean values (GMV) were calculated for each 2D dose plane comparison for the individual IMRT fields and VMAT arcs (268 comparisons). The GPR is the percentage of assessed points that have a gamma score of less than or equal to 1. The GMV is the mean of the gamma scores of all assessed points in the 2D distribution. Similarly GPR and GMV were calculated for the composite 3D dose distributions using 3D gamma analysis with both dose distributions interpolated to 0.4 times the distance-to-agreement metric.

A multivariable linear model was made for quantitative assessment of the significance/contribution of different (explanatory) variables on the overall outcome of the audit. This is a standard statistical technique to examine the influence of different variables on an overall result. Explanatory variables that were chosen were Linac-TPS combination, TPS calculation grid resolution, IMRT or VMAT delivery, age of EPID, treatment site (HN or PP), record and verification (R&V) system type and nominal dose-rate. The EPID to dose conversion method was developed using measured doses in water and EPID images from Varian Clinac linear accelerator for aS1000 type EPID [8] and Truebeam linear accelerators for aS1200 type EPID [10, 11] at a center with Eclipse planning system. Therefore this will examine whether the Varian and Truebeam combinations with Eclipse produce higher pass-rates than other combinations. The other variables were chosen based on available data from each center for the audit. The linear model was based on analysis of least squares of the GMVs for the 268 *2D dose planes* in the audit. The influence of the explanatory variables was studied through both visual and statistical assessment. The visual assessment was made by scatterplot of the audit GMVs versus each variable. The Tukey-Kramer honest significance test (HSD) and student’s t-test were used for assessment of the significance of the differences in results due to the explanatory variables. Statistical studies were performed in JMP software [13].

Finally, to assess the consistency of the VESPA audit with other reported audits, the results were compared with published results. To this purpose, the incidence ratio (IR) was calculated as the ratio of the observed GPR for the VESPA audit to the reference audit. Comparisons should be ‘stable’ if the range for the 95% confidence interval is ‘small’, i.e. < 0.5. The 95% confidence interval was calculated using:

$$ IR\pm 1.96\left(\frac{IR}{\sqrt{\left(\#\mathrm{of}\kern0.5em \mathrm{observed}\kern0.5em \mathrm{planes}\right)}}\right) $$

## Results

**Fig. 1:**
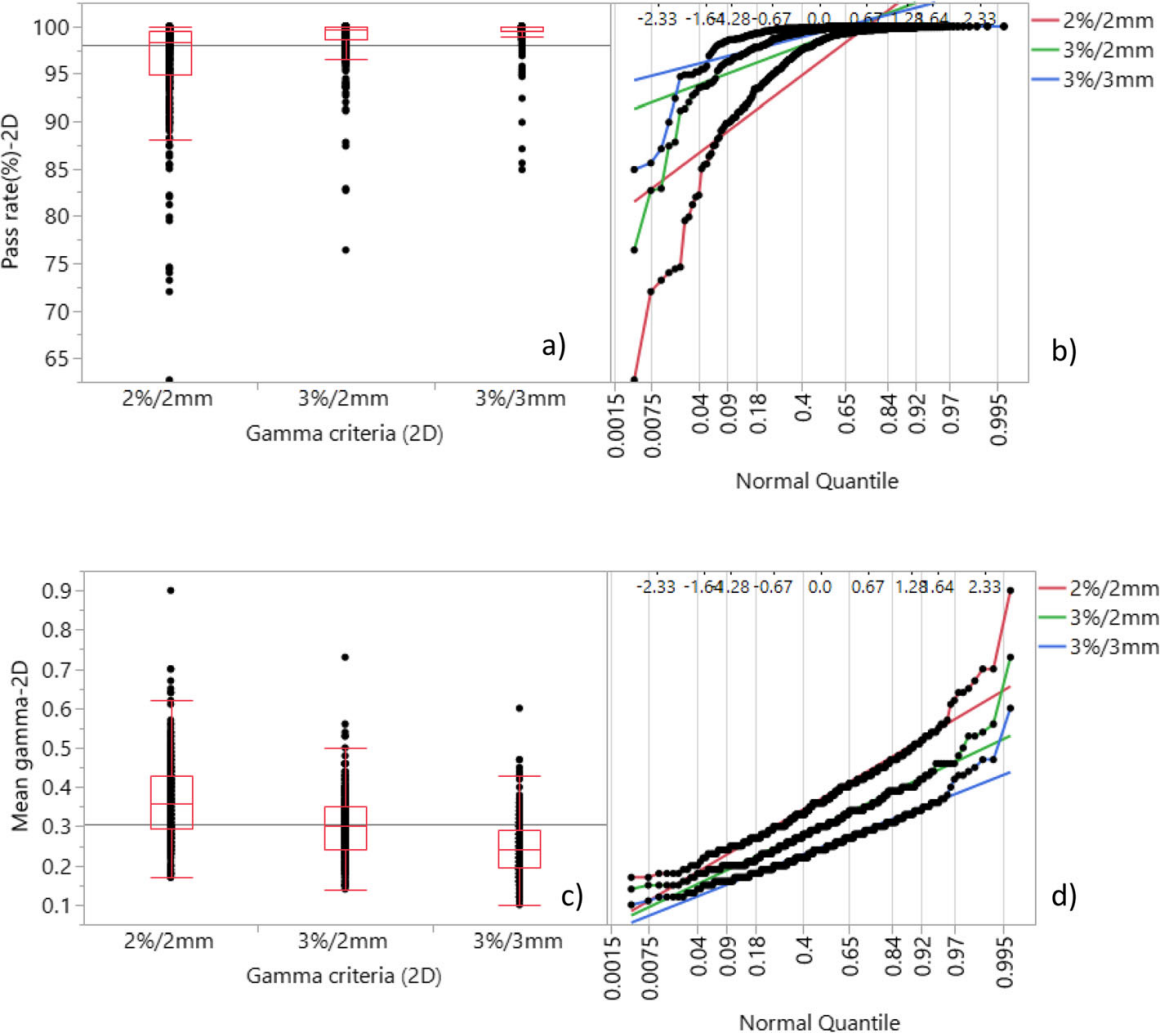
Gamma analysis results and normal quantile linearity for the 2D dose plane comparisons for 268 IMRT fields and VMAT arcs at 3%/3 mm, 3%/2 mm and 2%/2 mm criteria. The normal quantile linearity indicates normality of distributions. **a** GPRs; **b** GPR normal quantile; **c** GMVs; **d** GMV normal quantile

Figure 1(a) and (c) demonstrate the spread of GPRs and GMVs for different criteria for the measured planar IMRT fields and VMAT arcs. Maximum GPRs were 100.0% and minimum GPRs were 84.9%, 76.4% and 62.7% for 3%/3 mm, 3%/2 mm and 2%/2 mm criteria respectively. The mean GPRs and GMVs are shown in Table 1. Normal quantiles are plotted for both GPRs and GMVs in Fig. 1(b) and (d). As these figures suggest, more linearity is visually observed for GMV than GPR, indicating better normal distribution of GMV.

**Table 1:** Summary of the 2D audit gamma results

Gamma criteria	GPR (1 SD)	GMV (1 SD)
2%,2 mm	96.2 (5.5)%	0.37 (0.11)
3%,2 mm	98.6 (2.7)%	0.30 (0.09)
3%,3 mm	99.3 (1.9)%	0.25 (0.07)

**Fig. 2:**
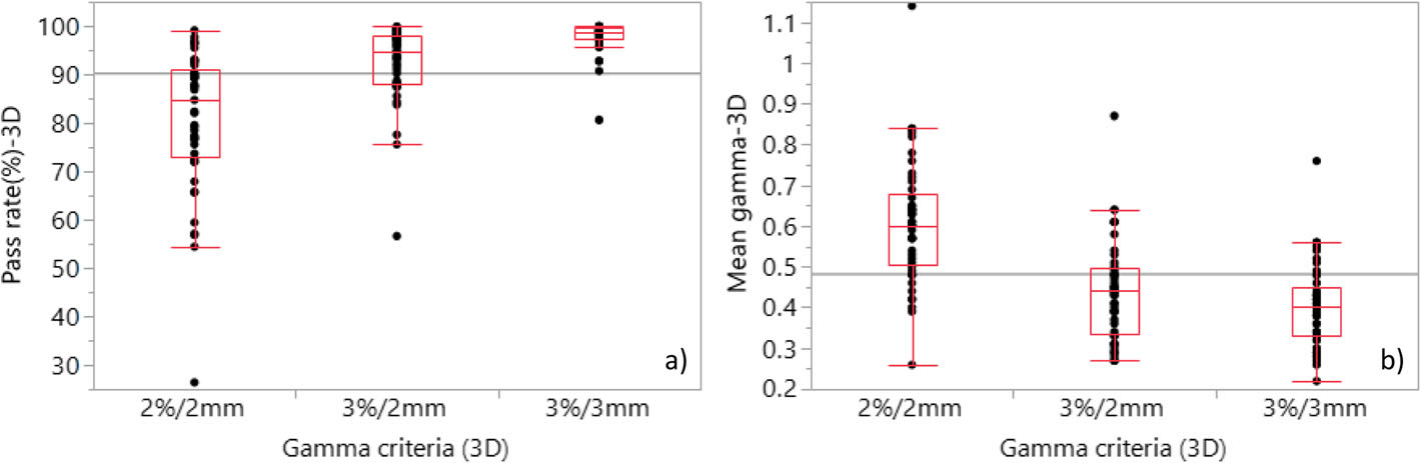
**a** GPRs and (**b**) GMVs for composite 3D dose analysis for the plans

The composite 3D dose distributions were analysed for the HN and PP plans in the VCP. Figure 2(a) illustrates the GPRs and Fig. 2(b) the GMVs for the 3D gamma analysis. The maximum GPRs were 100.0%, 99.9% and 99.1% and the minimum GPRs were 80.6%, 56.6% and 26.4% for 3%/3 mm, 3%/2 mm and 2%/2 mm criteria respectively. Mean GPRs (±1SD) were 97.7 (3.3)%, 92.5 (8.0)% and 80.8 (14.5)% for the same criteria.

A multiple variable linear model was made using the GMVs for the 2D dose plane comparisons. Table 2 summarizes the model outcome for the explanatory variables. The most influential variables in determining the results were Linac-TPS combination, TPS grid resolution and delivery type (IMRT or VMAT). The least significant variables were EPID age, treatment site, record and verification system and dose-rate.

**Table 2:** Effect of the explanatory variables on overall audit results. The columns have been ordered according the significance of each variable on the results

Variable	LogWorth	*p*
Linac - TPS	12.824	0.00000
TPS grid resolution	4.782	0.00002
IMRT/VMAT Delivery	3.855	0.00014
EPID age-5ys	2.030	0.00933
Treatment site	0.976	0.10561
R&V	0.814	0.15353
Dose rate	0.011	0.97501

**Fig. 3:**
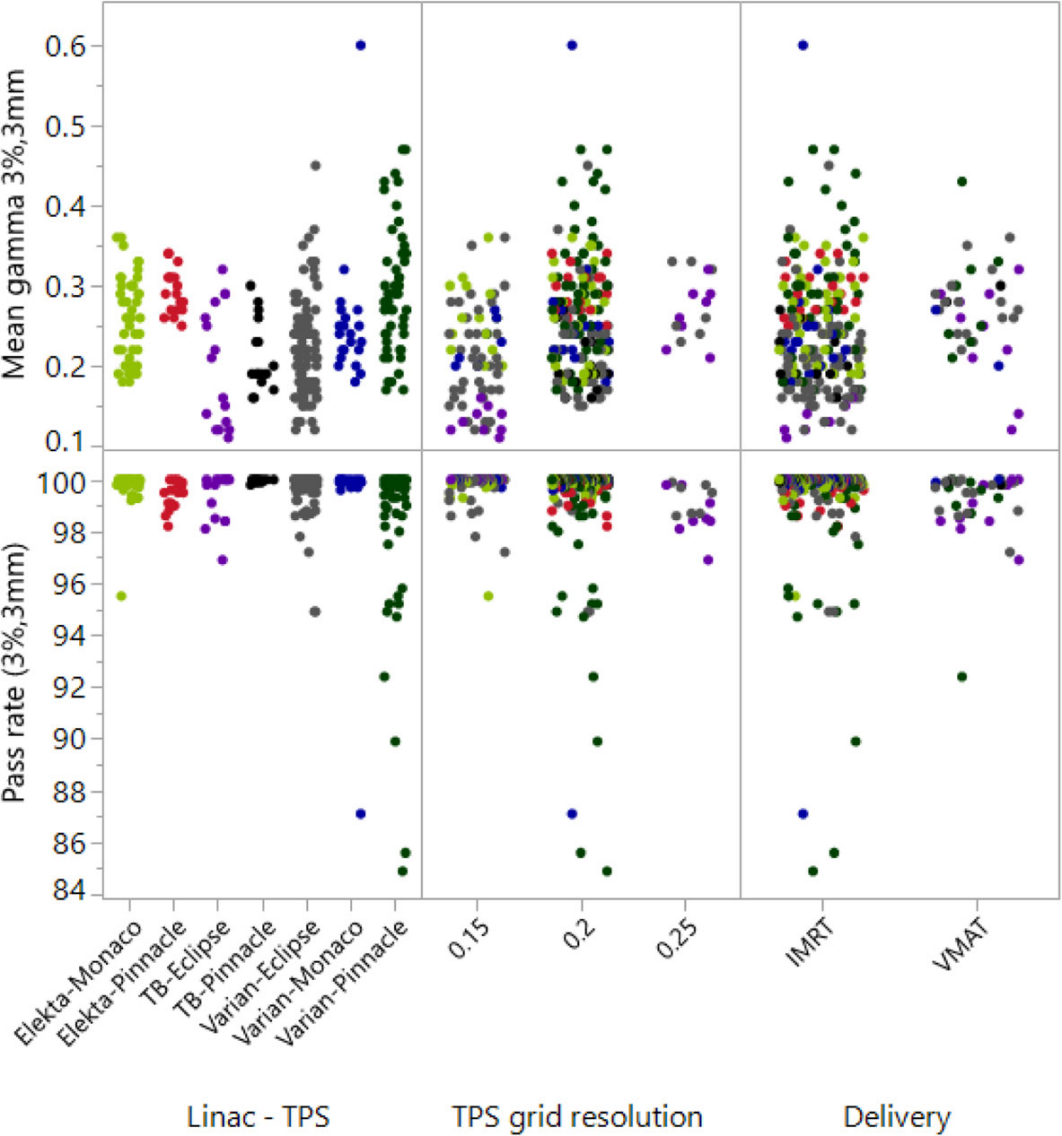
Scatterplot of the GMVs and the GPRs for the 2D dose plane comparisons of the audit versus the most significant explanatory variables (Linac-TPS combination, dose grid resolution and delivery type)

Figure 3 shows GPR and GMV scatterplots for the three most significant explanatory variables. The 1st plot for Linac-TPS combination shows some apparent distinctions between results for different combinations of linear accelerator and TPS type. The other two variables were colored according to the Linac-TPS combination.

**Fig. 4:**
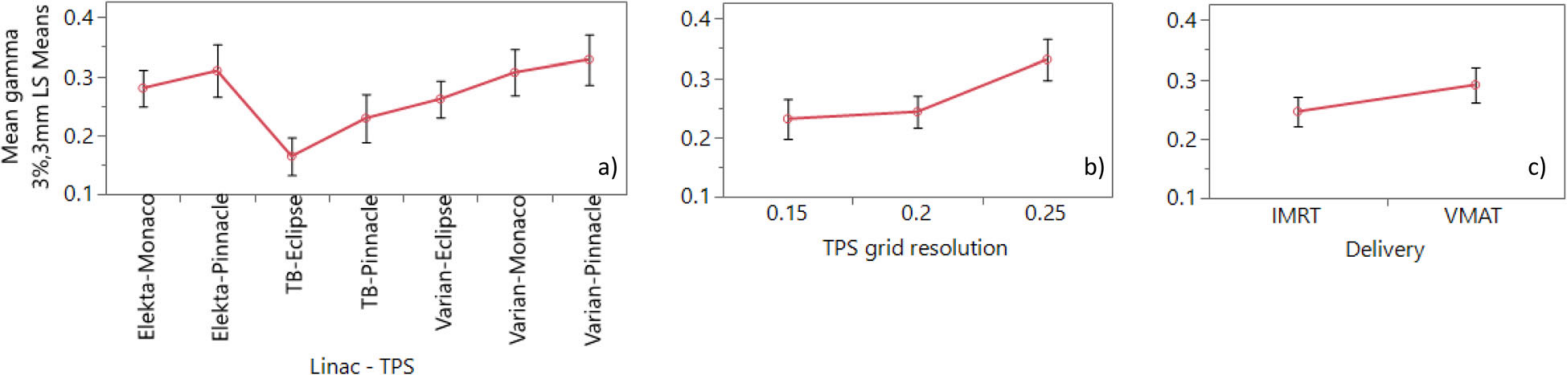
Plot of GMV for the three explanatory variables that showed most influence on the audit results (Linac-TPS combination, TPS dose grid resolution and IMRT/VMAT delivery)

Figure 4 contains plots of GMVs (3%, 3 mm) estimated marginal means (EMM, named lsmeans in JMP) and 95% CIs for the three variables with significant effects in the model. Follow-up testing of the significant differences (Tukey-Kramer HSD/student’s t test) between the means for the significant variables led to the following interpretations. For Linac-TPS, TB-Eclipse had a significantly lower mean than all other combinations (except TB-Pinnacle). The 4 combinations Elekta-Monaco, Elekta-Pinnacle, Varian-Monaco and Varian-Pinnacle were not significantly different to each other and appear to form a group with similarly high levels. There was some support for TB-Pinnacle and Varian-Eclipse having somewhat lower levels than the high group of 4 with 4 instances of significantly lower means (TB-Pinnacle lower than Varian-Monaco, Varian-Pinnacle and Elekta-Pinnacle, Varian-Eclipse lower than Varian-Pinnacle). For TPS grid, resolution 0.25 cm had higher GMV than the other two conditions which were both the same. For delivery, VMAT was higher than IMRT. Additional file 1 lists the test results.

Table 3 summarises the gamma comparisons at 2%/2 mm between VESPA and five conventional audits. Comparisons were made as variable specific as possible based on the published data, resulting in 16 comparisons. For the comparisons, 5 out of 16 were unstable as their interval range was quite ‘wide’, > = 0.5, and no conclusion was made for them. Among stable comparisons, 7 indicated similar pass rates of the VESPA with other audits (IR = 1) and 4 comparisons demonstrated higher pass rates for VESPA than the other audits (IR > 1).

**Table 3:** Comparison of the VESPA audit results with other recent audits. The GPRs are compared at 2%/2 mm criteria

Ref	Variable	Compare group *γ* GPR % (no)	VESPA study *γ* GPR % (no)	IR (95% CI)	Range	Significance/stability
1- [14]	Linac type	Median				
	Varian	96.7 (25)	96.8 (26)	1.0 (0.8–1.2)	0.4	Insignificant/stable
	TB		96.2 (12)	–		
	TPS type	Median				
	Eclipse	97.3 (22)	96.3 (26)	1.0 (0.8–1.2)	0.4	Insignificant/stable
	Monaco	98.8 (4)	98.5 (2)	1.0 (0.9–1.1)	0.2	Insignificant/stable
	Pinnacle	88.7 (6)	96.1 (10)	1.1 (1.0–1.2)	0.2	Significant/stable
2-[15]	Delivery type	Mean				
	IMRT	90.0 (23)	96.3 (230)	1.1 (0.9–1.3)	0.4	Significant/stable
	VMAT	93.0 (31)	95.5 (38)	1.0 (0.8–1.2)	0.4	Insignificant/stable
	TPS type	Mean				
	Eclipse	95.0 (21)	98.0 (113)	1.0 (0.8–1.2)	0.4	Insignificant/stable
	Monaco	84.0 (5)	96.4 (68)	1.1 (0.9–1.4)	0.5	Significant/unstable
	Pinnacle	91.7 (19)	93.7 (87)	1.0 (0.8–1.2)	0.4	Insignificant/stable
	Treatment site	Mean				
	H&N	90.0 (25)	95.2 (135)	1.1 (0.8–1.3)	0.5	Significant/unstable
	Pelvic	93.0 (10)	97.2 (133)	1.0 (0.8–1.3)	0.5	Insignificant/unstable
5-[21]	Delivery type	Mean				
	IMRT	92.0 (155)	96.3 (230)	1.0 (0.8–1.3)	0.5	Insignificant/unstable
6-[22]	IMRT/VMAT	Mean				
		90.0 (1265)	96.2 (268)	1.1 (0.9–1.3)	0.4	Significant/stable
7-[23]	VMAT	Mean				
		88.0 (118)	95.5 (38)	1.1 (0.9–1.3)	0.4	Significant/stable

## Discussion

The 3D composite dose audit results showed lower GPRs and larger GMVs than the 2D individual field/arc dose plane audit results. The 3D analysis could not currently be performed with 3%, 2 mm criteria as recommended by TG218 report for pre-treatment QA methods while the 2D analysis would meet this criteria. However the 3D analysis is sensitive to gantry angle errors as the dose for each image is calculated with the acquired gantry angle and is therefore an important component of the audit. The EPID measurement is inherently 2D and to estimate a 3D dose distribution in the virtual cylindrical phantom requires modelling of percentage depth dose. For the VESPA audit a single field percentage depth dose model was used which was also center independent. Improvement using a field-size specific and/or center-specific depth dose model could be explored. As a result the GMVs from the 2D individual field/arc dose comparisons were used for the statistical analysis in this paper.

Linac-TPS combination was found to influence the audit results. The Linac-TPS combination was used in the analysis rather than as separate variables due to the lack of spread of TPS type across all linac types which could bias results. The TB-Eclipse and TB-Pinnacle combinations were particularly found to result in lower GMV results. There are many potential reasons for this related both to this combination and the audit methodology. The Truebeam systems are a modern linac platform with high specifications for isocentre accuracy and other parameters. They have very accurate EPID positioning with active correction of EPID sag with gantry angle. The newer aS1200 imager does not have significant backscatter artifact which improves their performance for dosimetry. Plan complexity was not captured in the audit but could potentially have an effect. Future audits will include this parameter.

Centers were requested to produce the VFP and VCP plans at 0.2 cm or lower resolution although some submitted 0.25 cm resolution plans. The statistical analysis showed that the 0.25 cm resolution gave inferior results. The gamma algorithm used interpolates the TPS data to a high resolution to match the EPID resolution however this is clearly insufficient to counter the effect of the poor TPS resolution. Future audits will mandate the 0.2 cm or lower resolution based on these results. Another interesting finding was that the IMRT results showed lower GMV than the VMAT results. A possible explanation for this could be that the IMRT fields are acquired at fixed gantry angles and the data are corrected for EPID sag at these angles. However the VMAT acquires cine images during rotation and combines these into a single integrated image for 2D analysis. The individual cine images are not corrected for EPID sag and so the effect of this is likely to be greater and result in some blurring of the dose in the integrated image.

The VESPA audit is a TPS-planned audit not and end-to-end audit. These type of audits target a specific technology such as IMRT or VMAT and the CT scan of the phantom is typically provided to the center for planning. Comparing VESPA to other TPS-planned audits, the GPRs were similar to those from Clark et al. [14] for their audit of Varian VMAT deliveries conducted with the Octavius dosimetry system. While the VESPA results were higher for Elekta systems, the variability of these results meant that conclusions could not be drawn. For TPS systems the results were similar except for Pinnacle systems where the VESPA results had higher GPRs. For IMRT audits as well as the Monaco TPS system, significantly higher GPRs were found for VESPA compared to the ArcCheck based audit of Eaton et al. [15].

For the VESPA audit as for most audits and in-house pre-treatment quality assurance the pass/fail criteria were arbitrarily set. It was not possible to know the uncertainties in a particular centers’ TPS data or linac measurements. Pass/fail criteria could be set for future audits based on a statistical analysis of the current audit so that outlier centers could be identified. However the future audit would have to use a similar methodology and the same EPID to dose conversion model.

There are some limitations of this study. The measurement equipment are not completely standardized with differences between Varian EPID types (aS1000, aS1200) and Elekta imagers as well as equipment age. Data was collected on EPID response linearity as part of the study to ensure consistent results. 2D dose plane analysis is not ideal particularly for VMAT deliveries where a composite dose analysis would be preferred. By improving the 3D calculation model, it should be possible to audit centers using 3D dose distribution methods with more sensitive criteria (e.g. 3%, 2 mm). Another possibility is to use dose-volume-histogram methods where the ratio of 2D doses is backprojected through the benchmark CT plan and hence percentage depth dose modelling is not required. Elekta linacs are not currently audited for VMAT using VESPA due to difficulties in obtaining cine images and gantry angle information. This is possible with Elekta’s newer hardware and software (version 3.41) and a licence to access pixel scaling information, however these were not available at the time of the audit.

The EPID to dose conversion models were developed based on Varian Clinac and Truebeam measured beam data for single linacs and has been applied to multiple linacs of the same type. The model derived on Varian Clinac was applied to the Elekta linacs for this audit. Comparison of the field-size responses for the Elekta and Varian linacs using the TPS calibration plan data (2×2 to 25×25 cm^2^ fields) showed that there was a small difference in the average field size factor for the two linac types of maximum 2.1% for the smallest field, and average 0.6%. The field size factors from dose derived from Elekta images of the above fields compared to the TPS data showed greater differences than for Varian/TB centers’ data. The average of the absolute difference was 1.2% for Elekta and 0.6% for Varian/TB. Recently a model derived with Elekta measured data was compared to the Varian derived model for Elekta linacs in a separate study that has been submitted for publication. The improvement in results was small and not sufficient to affect the results of the current study.

Though not an end-to-end audit, the VESPA method provides a potentially inexpensive and rapid method to perform dosimetric auditing for specific assessments of new technologies. It takes about 2–4 h to do the planning and delivery. There are several commercial systems available that perform 2D planar EPID dosimetry including but not limited to PortalVision (Varian Medical Systems), Epiqa (Epidose), Epibeam (Dosisoft), and PerFraction (Sun Nuclear) [16, 17]. Some of these systems incorporate simple EPID support-arm backscatter corrections which apply to Varian R and E-Arm systems [18–20]. Currently the analysis is based on in-house software. This software has several advantages over commercial systems in that it has a sophisticated kernel-based backscatter correction; it accounts for EPID sag with gantry angle; and it allows 3D dose determination particularly for VMAT using cine-imaging. This software is not currently commercially available however the authors consider requests from users for the software wherever possible. In principle the VESPA method can be applied in exactly the same way to flattening filter free (FFF) beams however there are currently hardware limitations for imaging these high-dose rate beams on the older EPID systems that are still prevalent. The newer Varian and Elekta EPID systems have FFF imaging capability. The EPID to dose conversion method has also not to date been developed or benchmarked for small field dosimetry auditing.

## Conclusion

A new EPID-based remote dosimetric TPS-planned auditing method (VESPA) has been successfully applied to 30 audits of IMRT and VMAT for 21 centers across Australia and New Zealand. 2D dose-plane analysis was found to give more consistent results than 3D analysis. Statistical analysis of the results showed that there was some influence of Linac-TPS combinations on the results. This work shows that the remote EPID method can be used to audit centers with gamma pass-rates comparable or higher than other recent audits.

## Additional file

Additional file 1: **Table S1.** Participating centers in the VESPA audit and explanatory variables details for each center. **Table S2.** Statistical testing of the differences between audit results (GMV) for the explanatory variables. Results with asterisk indicate significant differences where Variable 1 (V1) has lower GMV than Variable 2 (V2). (DOCX 25 kb)

## Abbreviations

2D: Two-dimensional
3D: Three-dimensional
CI: Confidence interval
CT: Computed tomography
EPID: Electronic portal imaging device
GMV: Gamma mean value
GPR: Gamma pass rate
HN: Head and neck
HSD: Honest significance test
IMRT: Intensity modulated radiation therapy
IR: Incidence ratio
Linac: Linear accelerator
MLC: Multi-leaf collimator
PP: Post-prostatectomy
QA: Quality assurance
R&V: Record and verify
SSD: Source-surface distance
TB: Truebeam
TPS: Treatment planning system
TROG: Trans-Tasman Radiation Oncology Group
VCP: Virtual cylindrical phantom
VESPA: Virtual epid standard phantom audit
VFP: Virtual flat phantom
VMAT: Volumetric modulated arc therapy
