# Supplementary material for: A remote EPID-based dosimetric TPS-planned audit of centers for clinical trials: outcomes and analysis of contributing factors
Source: Radiat Oncol. 2018 Sep 17;13:178. doi: 10.1186/s13014-018-1125-8 (PMC6142693; doi:10.1186/s13014-018-1125-8)
Supplement: Supplementary file 1 — Table S1. Participating centers in the VESPA audit and explanatory variables details for each center. Table S2. Statistical testing of the differences between audit results (GMV) for the explanatory variables. Results with asterisk indicate significant differences where Variable 1 (V1) has lower GMV than Variable 2 (V2). (DOCX 25 kb) [file 13014_2018_1125_MOESM1_ESM.docx]

## Additional file 1

Table S1 Participating centers in the VESPA audit and explanatory variables details for each center.

| Center No | TPS-Linac | Grid Resolution (cm) | IMRT delivery type | | EPID age (5ys) | R&V | Treatment site | | | | Dose rate (MU/min) |
| --- | --- | --- | --- | --- | --- | --- | --- | --- | --- | --- | --- |
|  |  |  |  |  |  |  | **IMRT** | | **VMAT** | |  |
| 1 | Pinnacle-Varian | 0.20 | IMRT | - | 1 | Aria | HN | PP | - | - | 400 |
| 2 | Eclipse-Varian | 0.15 | IMRT | VMAT | 1 | Aria | HN | PP | HN | PP | 600 |
| 3 | Eclipse-Varian | 0.15 | IMRT | VMAT | 2 | Aria | HN | PP | HN | PP | 400 |
| 4 | Eclipse-Varian | 0.20 | IMRT | VMAT | 2 | Aria | HN | PP | HN | PP | 400 |
| 5 | Eclipse-Varian | 0.25 | IMRT | - | 2 | Mosaiq | - | PP | - | - | 400 |
| 6 | Eclipse-Varian | 0.20 | IMRT | - | 2 | Mosaiq | HN | PP | - | - | 400 |
| 7 | Pinnacle-TB | 0.20 | IMRT | VMAT | 1 | Mosaiq | - | PP | - | PP | 600 |
| 8 | Monaco-Varian | 0.15 | IMRT | VMAT | 2 | Aria | - | PP | HN | - | 600 |
| 9 | Pinnacle-Varian | 0.20 | IMRT | VMAT | 2 | Aria | - | PP | HN |  | 400 |
| 10 | Pinnacle-TB | 0.20 | IMRT | VMAT | 1 | Mosaiq | - | PP | - | PP | 600 |
| 11 | Pinnacle-Varian | 0.20 | IMRT | VMAT | 1 | Aria | HN | PP | HN | PP | 600 |
| 12 | Eclipse-TB | 0.15 | IMRT | VMAT | 1 | Mosaiq | - | PP | - | PP | 600 |
| 13 | Pinnacle-Elekta | 0.20 | IMRT | - | 1 | Mosaiq | HN | PP | - | - | 400 |
| 14 | Monaco-Elekta | 0.20 | IMRT | - | 1 | Mosaiq | HN | PP | - | - | 600 |
| 15 | Monaco-Elekta | 0.15 | IMRT | - | 1 | Mosaiq | HN | PP | - | - | 600 |
| 16 | Monaco-Elekta | 0.20 | IMRT | - | 1 | Mosaiq | HN | PP | - | - | 600 |
| 17 | Monaco-Varian | 0.20 | IMRT | VMAT | 2 | Mosaiq | HN | - | - | - | 600 |
| 18 | Eclipse-Varian | 0.20 | - | VMAT | 1 | Mosaiq | - | - | HN | - | 600 |
| 19 | Eclipse-TB | 0.25 | - | VMAT | 1 | Aria | - | - | HN | PP | 300 |
| 20 | Eclipse-TB | 0.25 | - | VMAT | 1 | Aria | - | - | HN | PP | 600 |
| 21 | Eclipse-Varian | 0.15 | - | VMAT | 1 | Aria | - | - | - | PP | 600 |

Table S2 Statistical testing of the differences between audit results (GMV) for the explanatory variables. Results with asterisk indicate significant differences where Variable 1 (V1) has lower GMV than Variable 2 (V2).

| Linac-TPS | | |  | TPS grid resolution | | |  | Delivery type | | |
| --- | --- | --- | --- | --- | --- | --- | --- | --- | --- | --- |
| V1 | V2 | p |  | V1 | V2 | p |  | V1 | V2 | p |
| Elekta-Monaco | Elekta-Pinnacle | 0.7933 |  | 0.15 | 0.20 | 0.5113 |  | IMRT | VMAT | 0.0001* |
| Elekta-Monaco | Varian-Pinnacle | 0.1888 |  | 0.15 | 0.25 | <.0001* |  |  |  |  |
| Elekta-Monaco | Varian-Monaco | 0.8233 |  | 0.20 | 0.25 | <.0001* |  |  |  |  |
| Elekta-Pinnacle | Varian-Pinnacle | 0.9718 |  |  |  |  |  |  |  |  |
| TB-Eclipse | Elekta-Monaco | <.0001* |  |  |  |  |  |  |  |  |
| TB-Eclipse | Elekta-Pinnacle | <.0001* |  |  |  |  |  |  |  |  |
| TB-Eclipse | TB-Pinnacle | 0.1057 |  |  |  |  |  |  |  |  |
| TB-Eclipse | Varian-Eclipse | <.0001* |  |  |  |  |  |  |  |  |
| TB-Eclipse | Varian-Monaco | <.0001* |  |  |  |  |  |  |  |  |
| TB-Eclipse | Varian-Pinnacle | <.0001* |  |  |  |  |  |  |  |  |
| TB-Pinnacle | Elekta-Monaco | 0.0632 |  |  |  |  |  |  |  |  |
| TB-Pinnacle | Elekta-Pinnacle | 0.0175* |  |  |  |  |  |  |  |  |
| TB-Pinnacle | Varian-Eclipse | 0.7148 |  |  |  |  |  |  |  |  |
| TB-Pinnacle | Varian-Monaco | 0.022* |  |  |  |  |  |  |  |  |
| TB-Pinnacle | Varian-Pinnacle | 0.0003* |  |  |  |  |  |  |  |  |
| Varian-Eclipse | Elekta-Monaco | 0.92 |  |  |  |  |  |  |  |  |
| Varian-Eclipse | Elekta-Pinnacle | 0.1113 |  |  |  |  |  |  |  |  |
| Varian-Eclipse | Varian-Monaco | 0.4193 |  |  |  |  |  |  |  |  |
| Varian-Eclipse | Varian-Pinnacle | <.0001* |  |  |  |  |  |  |  |  |
| Varian-Monaco | Elekta-Pinnacle | 1 |  |  |  |  |  |  |  |  |
| Varian-Monaco | Varian-Pinnacle | 0.9845 |  |  |  |  |  |  |  |  |
